# Modeling Top-Down and Bottom-Up Drivers of a Regime Shift in Invasive Aquatic Plant Stable States

**DOI:** 10.3389/fpls.2019.00889

**Published:** 2019-07-10

**Authors:** Emily F. Strange, Pietro Landi, Jaclyn M. Hill, Julie A. Coetzee

**Affiliations:** ^1^Centre for Biological Control, Department of Zoology and Entomology, Rhodes University, Grahamstown, South Africa; ^2^Theoretical Ecology Group, Department of Mathematical Sciences, Stellenbosch University, Matieland, South Africa; ^3^Centre for Biological Control, Department of Botany, Rhodes University, Grahamstown, South Africa

**Keywords:** floating macrophytes, submerged macrophytes, invasion, biological control, resilience

## Abstract

The evidence for alternate stable states characterized by dominance of either floating or submerged plant dominance is well established. Inspired by an existing model and controlled experiments, we conceptually describe a dynamic that we have observed in the field using a simple model, the aim of which was to investigate key interactions of the shift between invasive floating and invasive submerged plant dominance, driven by the rapid decomposition of floating plants as a consequence of herbivory by biological control agents. This study showed that the rate of switch between floating and submerged invasive plant dominance, and the point in time at which the switch occurs, is dependent on the nutrient status of the water and the density of biological control agents on floating plant populations. Therefore, top-down invasive plant biological control efforts using natural enemies can affect systems on a wider scale than the intended agent – plant level, and can be significantly altered by bottom-up changes to the system, i.e., nutrient loading. The implications of this are essential for understanding the multiple roles invasive plants and their control have upon ecosystem dynamics. The results emphasize the importance of multi-trophic considerations for future invasive plant management and offer evidence for new pathways of invasion. The model outputs support the conclusion that, after the shift and in the absence of effective intervention, a submerged invasive stable state will persist.

## Introduction

Regime shifts in ecological systems can occur rapidly and suddenly, causing changes in key structures and functioning that can threaten sustainability and be difficult to reverse (Scheffer et al., 2003; MacNally et al., 2014; Rocha et al., 2015). These shifts, such as switches in lakes from clear water to algal blooms, can result from relatively small changes in environmental pressures but once a critical threshold is passed, the key mechanisms maintaining the system are disrupted or broken, altering the system trajectory toward a new regime. New feedback mechanisms then develop, allowing the new regime to become stable (Beisner et al., 2003; Scheffer and Carpenter, 2003; Folke et al., 2004; Walker and Meyers, 2004; Kinzig et al., 2006; Biggs et al., 2009).

The existence of alternate stable states with basins of attraction dominated by floating and submerged plant species is a classic example of a regime shift and is well documented in freshwater lakes, supported experimentally, observationally, and theoretically (Scheffer et al., 2003; Folke et al., 2004; Netten et al., 2010). Scheffer et al.’s (2003) seminal paper presents a mathematical model describing the key interactions among the main variables. The model, although contextually broad, explores the asymmetry between floating and submerged plant stable states with regards to their competition for resources, where submerged plants are able to access nutrients in the sediment not available to the floating plants, but are less able to compete for light (Scheffer, 2009). The switch between states can occur rapidly, and the subsequent changes in aquatic plant community structures have trophic cascade effects, resulting in altered water, sediment, and nutrient cycling regimes (Blindow et al., 1993; Yarrow et al., 2009; Havel et al., 2015).

Invasive macrophytes, whose establishment and spread continues to be one of the leading threats to global freshwater ecosystems, significantly alter ecosystem structure and functioning whilst limiting access to vital ecosystem services (Lovell et al., 2006; Hussner et al., 2017). South Africa, in particular, has been heavily impacted by floating invasive macrophytes such as water hyacinth (*Eichhornia crassipes* Mart. Solms (Pontederiaceae) and water lettuce [*Pistia stratiotes* L. (Araceae)], which form dense mats on the water’s surface as a result of nutrient loading, release from natural enemies, and a relatively small native macrophyte species diversity with which to compete (Coetzee et al., 2011b). These mats reduce biodiversity, limit access to potable freshwater, increase both siltation of rivers and flood risks, drown livestock and damage vital infrastructure (Janse and Van Puijenbroek, 1998; Scheffer et al., 2003; Caraco et al., 2006). Classical biological control (CBC) initiatives using host specific natural enemies have successfully reduced many of these invasions to the extent they are now regarded as being under control (Hill and Coetzee, 2017).

The past decade has seen an increase in the establishment of multiple invasive submerged plant species following the control of floating macrophytes, which is a major concern for the future safeguarding of South Africa’s freshwater (Hill and Coetzee, 2017). Notorious submerged invasive species such as *Myriophyllum spicatum* L. (Haloragaceae), *Hydrilla verticillata* (L.F.) Royle (Hydrocharitaceae) and *Egeria densa* Planch. (Hydrocharitaceae) have successfully established far more widely than previously thought (Madeira et al., 2007; Coetzee et al., 2011b; Martin and Coetzee, 2011; Weyl and Coetzee, 2014). Globally, the biological control programs associated with floating or emergent macrophytes have been highly successful, but similar biological control of submerged plant species has proved more challenging (Schmitz and Schardt, 2015). For example, the first biological control agent against *H. verticillata* was released in the United States in 1988 and over a quarter of a century later, it is still considered the most problematic aquatic plant in the United States (Gu, 2006; True-Meadows et al., 2016).

**FIGURE 1:**
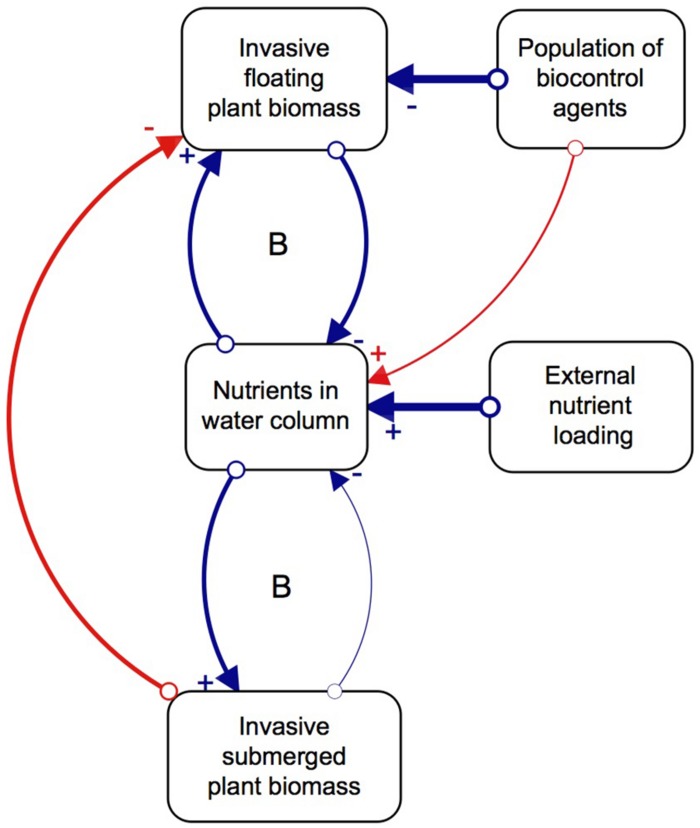
Direct (blue) and indirect (red) relationships between key factors and processes of the regime shift, and the feedback mechanisms that balance (B) the system. Created in STELLA Professional (iSEE systems Inc., Version 1.0.3). The strength of interactions is depicted by the thickness of the arrows connecting the variables that have a positive (+) or negative impact on the other (–). Revised from Strange et al. (2018).

Although biological control has effectively reduced populations of floating invasive plants, the effect this has on the submerged plant community structure is relatively unknown. The majority of biological control programs traditionally investigate the direct interactions between a potential agent and its target species, while plant interaction experiments focus on changes within a single trophic level (Van et al., 1999; James et al., 2006; Martin and Coetzee, 2014). However, studying the indirect effects of the agents on the competitive interactions of the target species, as well as multitrophic cascading effects of biological control, would paint a more holistic picture of the impacts they can have on a system (Harvey et al., 2010). We thus propose that, as floating invasive plants decompose due to herbivory pressure from biological control agents, nutrients, light, and space become available to submerged plants, which successfully capitalize on this new abundance of resources and proliferate (Chimney and Pietro, 2006; James et al., 2006; Shilla et al., 2006; Longhi et al., 2008). However, the relative paucity of native submerged plant species, as a result of few natural freshwater systems in the South African landscape, combined with external nutrient loading, means that invasive submerged plants are more likely to establish than native ones. Once the invasive submerged plants are established, their ability to rapidly grow and capitalize on available nutrients allows them to dominate the system (Szabo et al., 2010). In other words, the system has two basins of attraction, one dominated by floating invasive plants and the other by submerged invasive plants, where biological control induces the shift in dominance (Figure 1; Strange et al., 2018). The interactions between the three key variables of floating plants, nutrients and agents results in more favorable conditions for invasive submerged plant communities which lock up the available nutrients in the system, sustained by continued external nutrient loading.

**FIGURE 2:**
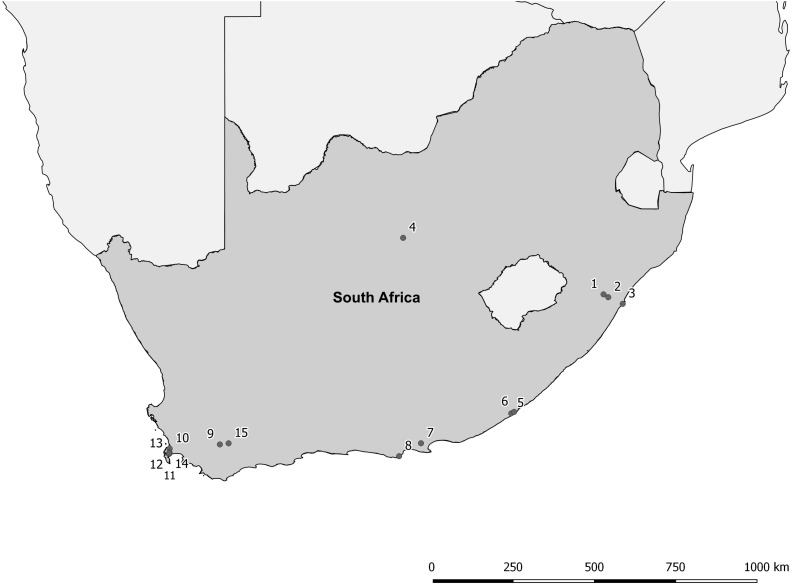
Distribution map of locations where a switch from floating invasive to submerged invasive plant dominance has been observed following biological control of the floating plant species (Coetzee, unpublished data, from Rhodes University annual aquatic weed surveys conducted from 2008 to 2015). For details of each site see Table 1.

The proposed shift is supported by existing theories on ecosystem invasibility such as the fluctuating resource hypothesis of invasion that assumes plant communities become more susceptible to invasion following increased availability of unused resources (Davis et al., 2000). Beyond theoretical support, we have documented multiple cases in the field of the shift in dominance from floating to submerged invasive plants following successful biological control of numerous floating species across South Africa (Table 1 and Figure 2). To further support these field observations, we explored the competitive interactions and relationships between three species in controlled mesocosm experiments, each representing the potential dominant states in a South African context; the floating invasive *P. stratiotes*, the submerged invasive *E. densa*, and the confamilial, trophically analogous native *Lagarosiphon major* Ridl. Moss ex Wager (Hydrocharitaceae). Differences in the responses of the native and non-native submerged species to the biological control of the floating plants, using the *P. stratiotes* control agent, *Neohydronomus affinis* Hustache (Coleoptera: Curculionidae) supported the hypotheses of nutrient loading and biological control acting as key drivers between states (Strange, 2017; Strange et al., 2018).

**TABLE 1:** Site numbers (corresponding to **Figure 2**), names and coordinates where a switch from floating invasive to submerged invasive plant dominance has been observed in the field following biological control of floating plant species (Coetzee, unpublished data, from Rhodes University annual aquatic weed surveys conducted from 2008 to 2015).

**No.**	**Site name**	**Coordinates**	**Floating spp.**	**Control agent(s)**	**Submerged spp.**
1	Riverlea, Ashburton	−29.676780, 30.462460	*Salvinia molesta*	*Cyrtobagous salviniae*	*Egeria densa*
2	Cato Ridge Golf Course, Cato Ridge	−29.754497, 30.593318	*S. molesta*	*C. salviniae*	*E. densa*
3	Bluff Nature Reserve, Durban	−29.938398, 30.992749	*S molesta*	*C. salviniae*	*Ceratophyllum demersum*
4	Vaalharts Weir, Warrenton	−28.114557, 24.927286	*Eichhornia crassipes*	*Neochetina eichhorniae; N. bruchi*	*Myriophyllum spicatum*
5	Nahoon River, East London	−32.964137, 27.913206	*E. crassipes*	*N. eichhorniae; N. bruchi; Eccritotarsus catarinensis*	*E. densa*
6	Etna Farm Dam, Gonubie	−32.924842, 27.993539	*Pistia stratiotes*	*Neohydronomus affinis*	*C. demersum*
7	Swartkops River, Port Elizabeth	−33.790993, 25.420586	*E crassipes*	*N. eichhorniae; N. bruchi*	*E. densa*
8	St Francis Marine, Cape St Frances	−34.148490, 24.815530	*S. molesta*	*C. salviniae*	*E. densa*
9	Breede River, Robertson	−33.823270, 19.865260	*S. molesta*	*C. salviniae*	*C. demersum*
10	Liesbeeck River, Cape Town	−33.93942, 18.47841	*E. crassipes*	*N. eichhorniae; N. bruchi*	*E. densa*
11	Mocke River, Cape Town	−34.044140, 18.474640	*E. crassipes*	*N. eichhorniae; N. bruchi*	*C. demersum*
12	Zandvlei, Cape Town	−34.085306, 18.461542	*E. crassipes*	*N. eichhorniae; N. bruchi*	*C. demersum*
13	Westlake River, Cape Town	−34.081266, 18.455327	*P. stratiotes*	*N. affinis*	*C. demersum*
14	Keyser’s River, Cape Town	−34.066997, 18.460870	*P. stratiotes*	*N. affinis*	*C. demersum*
15	Kogmanskloof River, Montagu	−33.793216, 20.105881	*S. molesta*	*C. salviniae*	*C. demersum*

Mathematical models of ecological systems cannot incorporate the full scope of natural processes, and compromises are made regarding spatial or temporal elements, but they are still useful for disentangling the individual mechanisms that lead to overall system changes (Bulling et al., 2006; Scheller et al., 2010; Chatzinikolaou, 2013). Subsequently, the aim of this study was to develop a qualitative, dynamic model that might offer initial theoretical support for a shift from invasive floating to invasive submerged macrophyte dominance, based on the hypothesis that nutrient loading and the application of biological control are the main drivers of species dominance.

## Materials and Methods

### Study Species

*Pistia stratiotes* was targeted for biological control in the 1980s with the introduction of the weevil *N. affinis* following the success of this control method in Australia (Harley et al., 1990). This species was chosen as the model invasive floating macrophyte due to the short timeframes required to achieve total control by herbivory from the agent (Coetzee et al., 2011b). Further still, a switch in states from dominance of *P. stratiotes* to submerged invasive macrophytes has been observed in a number of locations across South Africa as a result of biological control (Figure 2 and Table 1). *Egeria densa* was selected as the invasive submerged species as it has been identified as the most widespread submerged aquatic invader in South Africa (Coetzee et al., 2011a; Smith et al., 2019) and has been recorded in multiple sites where floating invaders previously dominated (Figure 2 and Table 1).

### Model

The following equations were developed, based on Scheffer et al.’s (2003) original model, to describe the main interactions emerged from previous experiments (Strange, 2017; Strange et al., 2018) between the key variables of the proposed regime shift between floating and submerged species, driven by biological control:

$$\frac{d\,F}{d\,t}=-l_{F}\,F+a_{F}\,N\,F-c_{F}\,\left(N\right)\,F^{2}-B\,F$$

$$\frac{d\,S}{d\,t}=-l_{S}\,S+a_{S}\,N\,S-c_{S}\,\left(N\right)\,S^{2}$$

where the changes over time (*t*) of the biomass of invasive floating plants (*F*) and invasive submerged plants (*S*) were modeled as a function of their mortality rates, *l*_*F*_ and *l*_*S*_, modified by the rates of nutrient uptake, *a*_*F*_ and *a*_*S*_, and their intraspecific and nutrient-dependent competitive abilities, *c*_*F*_⁢(*N*) and *c*_*S*_⁢(*N*). This dynamic is based on multiple studies demonstrating the impact of nutrients on intra- and interspecific competition of submerged (e.g., Martin and Coetzee, 2014; Strange, 2017; Strange et al., 2018) and floating (Njambuya et al., 2011) macrophyte populations. In the present model intraspecific competitive abilities *c*_*F*_⁢(*N*) and *c*_*S*_⁢(*N*) were modeled using the following equations:

$$c_{F}\,\left(N\right)=c_{F\,0}\,e\,x\,p\,\left(-e_{F}\,N\right)$$

$$c_{S}\,\left(N\right)=c_{S\,0}\,e\,x\,p\,\left(-e_{S}\,N\right)$$

where intraspecific competition decreases with available nutrients in the water column, *e*_*F*_ and *e*_*S*_represent the strength of this decay, and *c*_*F0*_ and *c*_*S0*_ the maximum competition. Interspecific competition is modeled through different uptake rates (*a*_*F*_ and *a*_*S*_) and different effect on nutrients in the water column, *m*_*F*_ and *m*_*S*_. In fact, available nutrients in the water column*N* changes with floating and submerged plant biomass dynamics according the following equation:

$$N=N_{0}-m_{F}\,\left(F-F_{0}\right)-m_{S}\,\left(S-S_{0}\right)$$

where *N*_0_,*F*_0_, and *S*_*0*_ are, respectively, the initial nutrient availability, floating plant, and submerged plant biomass. The effect of biological control, *B*, was incorporated into the model for floating plants as an additional mortality rate.

The parameters for the model (Table 2) were qualitatively chosen using a combination of the trends observed experimentally (Strange et al., 2018) and from the literature of the original floating to submerged shift model (Scheffer et al., 2003). Model dynamics are robust to the specific values chosen. Each plant population began with the same initial biomass. Mortality rates were assumed to also be equal as there are no specific data indicating otherwise (Scheffer et al., 2003). The floating plants were given a higher value for intraspecific competition compared to the submerged plants, as reflected in the results of previous experiments due to competition for light and space (Strange, 2017). Submerged plants are very efficient in locking up nutrients from the water column thus were assigned a higher value for such effect (Barko et al., 1988; Chen and Barko, 1988; Rattray et al., 1994; Mazzeo et al., 2003; Scheffer et al., 2003). The rate of nutrient uptake was set to be higher for floating plants than submerged as floating plants are better competitors for light, which increases plant growth and affects the size of roots, providing more surface area for nutrient uptake (DeBusk et al., 1981; Akinbile and Yusoff, 2012). In the experiments upon which the observations were made, nutrients (manipulated at different levels within the mesocosms in the form of NH_4_NO_3_) were determined to be a key driver (Strange et al., 2018) and the model was developed specifically to explore the interrelationship between the model species, biological control and nutrients, thus other environmental variables such as light were not directly modeled.

**TABLE 2:** Summary of model variables and parameters, their definitions and dimension.

**Parameter**	**Description**	**Dimension**
*F*	Biomass of floating invasive plant	Biomass
*S*	Biomass of submerged invasive plant	Biomass
*N*	Availability of nutrients in the water column	Mass
*B*	Biological control mortality rate	1/Time
*e_F*	Relationship of nutrients and competition (floating)	1/Mass
*e_S*	Relationship of nutrients and competition (submerged)	1/Mass
*c*_*F0*_	Intraspecific competition (floating)	1/(Biomass Time)
*c*_*S0*_	Intraspecific competition (submerged)	1/(Biomass Time)
*l_F*	Natural mortality rate (floating)	1/Time
*l_S*	Natural mortality rate (submerged)	1/Time
*a_F*	Rate of nutrient uptake (floating)	1/(Mass Time)
*a_S*	Rate of nutrient uptake (submerged)	1/(Mass Time)
*m_F*	Effect on nutrients (floating)	Mass/Biomass
*m_S*	Effect on nutrients (floating)	Mass/Biomass

The model was implemented within the STELLA^®^ Professional software environment (iSEE systems Inc., Version 1.0.3). STELLA models use stocks, flows and converters to produce time-series simulations. The model outputs trace the temporal changes in populations (stocks), as effected by the external pressures and parameters (converters), according to the equations which are used to describe their interactions (flows). Values were assigned to each stock (*F*_*0*_, *S*_*0*_, and *N*_*0*_), which provides the initial populations, and to each converter, which provides parameter values. The flows contain the model equations describing interactions and therefore have no numeric values.

## Results

**FIGURE 3:**
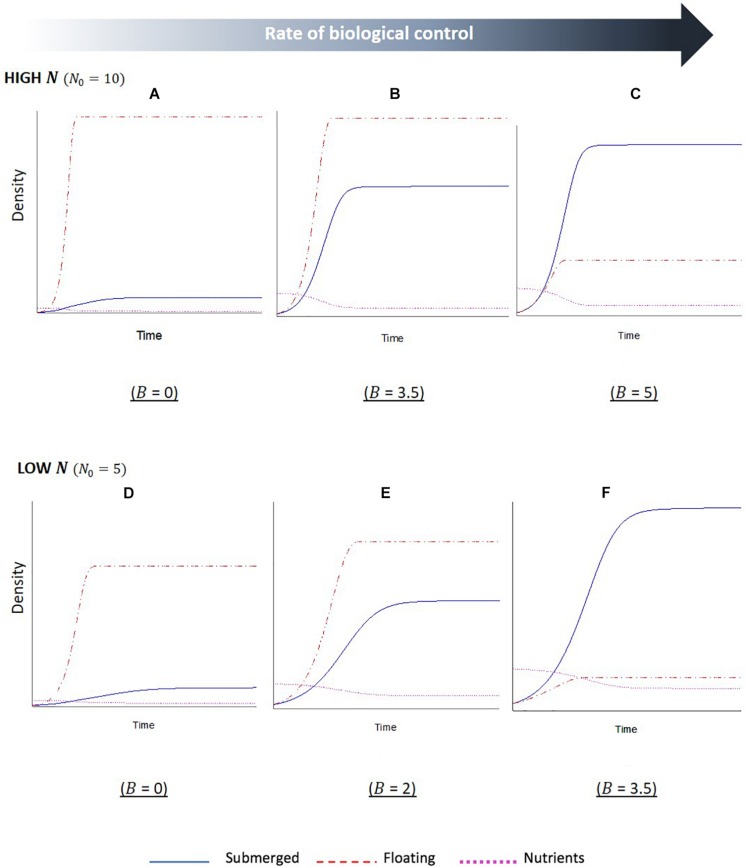
Stella simulation outputs showing temporal changes in floating (*F*, red dashed line) and submerged (*S*, blue solid line) plant biomass and nutrient (*N*, magenta dotted line) levels in scenarios with **(A–C)** high initial nutrients and **(D–F)** low initial nutrients. Within each nutrient condition, the rate of biological control (*B*) was altered, as indicated beneath each graph. Scales are not fixed. Initial conditions: *F*_0_ = *S*_0_ = 1. Parameter values: *l*_*F*_ = *l*_*S*_ = 1,*a*_*F*_ = 1,*a*_*S*_ = 0.5,*c*_*F*⁢0_ = 1,*c*_*S*⁢0_ = 0.5,*e*_*F*_ = *e*_*S*_ = 1,*m*_*F*_ = 0.01,*m*_*S*_ = 0.1.

The simulation outputs show temporal changes in the biomass of floating and submerged plant populations, with increased biomass indicating plant dominance, as well as changes in the levels of nutrients within the water column. For the first series of simulations, the initial starting level of nutrients was set to a relative high value (Figures 3A–C). The first output from this simulation (Figure 3A), where the biological control rate of the floating plants was set to zero, shows a sharp initial increase followed by a plateau in biomass of the floating plants. Whilst the submerged plants also initially increased in biomass, the growth was slower and plateaued a short while after the point in which the floating plant population peaked. This indicates the floating plants become dominant and are able to maintain dominance in the system. The second output of this simulation (Figure 3B), where nutrients remained high but the biological control rate was increased, reveals the same overall trend of floating plant dominance, but the relationship between the two plant biomass changed. There is a longer initial period of increased nutrients in the system (a product of the biological control of the floating plants and subsequent senescent plant material), and the submerged plant population demonstrates a much sharper increase before leveling off. The final output of this simulation (Figure 3C) shows a complete switch between the plant populations; as the biological control slows the growth of the floating population and increases the levels of nutrients in the system, there is an initial phase of co-existence between the plant populations. Eventually a point is reached, where the floating plants are completely limited by the biological control, and the submerged plant population is able to grow to a point that surpasses the floating plant biomass and maintain dominance.

The second series of model simulations (Figures 3D–F) shows outputs for systems, where the initial level of nutrients in the system was set to a lower initial value. The three outputs were once again set to a sequential increase in the extra mortality by biological control agents on the floating plants. The results show the same overall relationship between the floating and submerged plant populations, with a switch in dominance once a critical level of biological control agents has been crossed. However, there are some differences in the interactions. Firstly, in the reduced nutrient setting of these simulations the switch occurs less suddenly, with reduced slopes, indicating a slower rate of change for both plant populations. Secondly, the required rates of biological control that led to a subsequent shift in plant dominance were reduced in the lower nutrient setting. Lastly, there is a reduced disparity between the two plant biomasses when the biological control rates are at the lowest and highest setting compared to the high nutrients.

## Discussion

This model estimates, for the first time, the effects that current species-level management of floating invasive plants have upon wider community-level interactions, in a South African context, supporting the hypothesis that the switch between floating invasive and submerged invasive plant dominance can be influenced by the biological control of floating plants. While bottom-up driven changes to plant-herbivore interactions in aquatic systems have been demonstrated in multiple cases (e.g., Coetzee and Hill, 2012; Maseko et al., 2019), the model also supports the theory that top-down pressures (that affect ecosystems on a wider scale than the intended control agent and target plant level) can be significantly altered by bottom-up changes to the system (nutrient loading).

Reduced nutrient loading significantly increased the success of biological control of water hyacinth (*E. crassipes*) (Heard and Winterton, 2000; Coetzee and Hill, 2012); as less nutrients were available, macrophytes were not able to recover as quickly from herbivory damage (McNaughton, 1983), therefore plant mortality can be achieved at a lower density of biological control agents. This pattern was reflected in our model system outputs; the level of biological control required to alter plant dominance was reduced in scenarios where initial nutrient loading was lower. Center and Dray (2010) explored the effects of nutrient loading on the relationship between water hyacinth (*E. crassipes)* and two associated biological control agents (*Neochetina eichhorniae* Warner and Neochetina *bruchi* Hustache). Their results showed population growth for both agents was affected by plant quality; plants grown in high nutrient conditions were superior hosts for *N. bruchi* and there were significant increases in reproductive outputs of *N. bruchi*. They conclude that previously developed models aiming to simulate biological control of *E. crassipes* fell short because bottom-up drivers were under-estimated and overlooked. Our study supports this viewpoint and both underline how wider understanding of multi-trophic dynamics, explored using theoretical models can be applied to the practical aspects of invasive species control. Center and Dray (2010) present experimental data to develop a conceptual model to can aid future integrated invasive plant management strategies; as models become more accessible to a wider range of practitioners through more user-friendly interfaces, their role in applied decision making will inevitably become more prominent (Plagányi, 2007; García-Llorente et al., 2008; McCallum, 2008; Chatzinikolaou, 2013). Rightfully, there remain reservations regarding the application of modeling outputs to real life scenarios, such as over simplification of complex systems which highlight the importance of controlled and field-based experiments to describe the finer mechanisms of system changes and increase model validity.

Schroder et al. (2005), in reviewing the direct evidence for alternate stable states, concluded that future research in the field should focus primarily on the specific mechanisms behind switches in ecological states. Manipulation experiments may be bound by spatial and temporal constraints, but small-scale experiments can be crucial to help explain large-scale patterns, and can be a powerful way to show that a system has alternate attractors (Scheffer and Carpenter, 2003; Benton et al., 2007). Although Schroder et al. (2005) report a bias in the literature toward laboratory experiments, there is a paucity in multi-trophic experiments within the field of invasive plant research overall (Harvey et al., 2010; Villamagna and Murphy, 2010; Schultz and Dibble, 2012), yet they are essential for understanding internal ecosystem processes and they have been labeled as an over-looked asset in the exploration of regime shifts (Chase, 2003; Anderson et al., 2009). The validity of evidence claiming to support the existence of multiple stable states has been the subject of increased scrutiny and debate (Schroder et al., 2005; MacNally et al., 2014; Capon et al., 2015). Capon et al. (2015) argue that empirical field-based studies are severely lacking and report common false associations between theoretical constructs with results that do not support them. In agreement with this review, we recognize that whilst the model we present is neither predictive nor quantitative, it offers insight into the multitrophic consequences of invasive species control and the interplay between bottom-up and top-down drivers of ecosystem change. It is now crucial to identify whether or not the switch between floating invasive and submerged invasive plant dominance, instigated by biological control, is occurring in the field. If so, future studies are crucial to determine whether the management of these systems can be executed in a way that might reduce the likelihood of this shift, whilst increasing system resilience; and whether increasing native submerged plant populations prior to floating macrophyte removal could curb invasive submerged plant establishment. Restoration studies, where community assemblies are purposefully altered by selecting native species determined by resource-use traits to occupy vulnerable systems, have been shown to increase resistance of a community to successful invasion (Funk et al., 2008), and following the results of this study, indicate that this should be a priority to managers of invaded aquatic systems.

The efforts of this study aimed to bring together the theoretical concepts of alternative stable states and community change with the practical and applied domain of invasive species management and control. As with all models there are limitations that must be taken into consideration when drawing conclusions. The model is not quantitative and as such cannot be used as a predictive or diagnostic tool (and indeed this was not the intention). Future experiments could be used to develop and further parameterize the model by including more variables we know to be important in plant community structure such as light, water chemistry and presence of decomposers. The original experiments upon which the model was based used only the species described in this manuscript and exploring the same competitive dynamics between other plant species (of differing growth forms, families, etc.) would be both valid and interesting. The model was developed to offer initial theoretical support, along with field and laboratory-based observations, to the proposed multi-trophic consequences of floating invasive species control. In light of this the results presented do have the potential to better inform management of South Africa’s freshwater systems and highlight the benefit of continuing multi-trophic considerations for future invasive plant management worldwide, as well as opening up a multitude of possibilities for research into the mechanisms of submerged plant invasions and resilience of native macrophyte communities in South Africa, and further afield. Based on the findings presented in this manuscript, we recommend further investigations to increase understanding of the multi-trophic consequences of invasive species control and removal. Further, and more specifically to aquatic macrophyte invasions, we recommend a more holistic approach to the management of floating invasive plants including commitment to nutrient amelioration and post-control community restoration.

## Data Availability

The datasets generated for this study are available on request to the corresponding author.

## Author Contributions

ES conducted the experimental studies whose results were used for the simulation models. PL and ES developed and refined the models. JH and JC conceptualized and supervised the work, and commented on and edited the manuscript.

## Conflict of Interest Statement

The authors declare that the research was conducted in the absence of any commercial or financial relationships that could be construed as a potential conflict of interest.
